# Designing Rosie the Chatbot with and for Pregnant and New Mothers of Color:  a Community-Engaged Study Leveraging Artificial Intelligence and Prevention Science to Improve Maternal and Child Health Outcomes

**DOI:** 10.1007/s11121-026-01901-7

**Published:** 2026-04-17

**Authors:** Elizabeth M. Norell, Amara Channell Doig, Michelle Jasczynski, Alexis S. Hunter, Francia Ximena Marin Gutierrez, Heran Mane, Sourabh Mane, Xiaohe Yue, Quynh C. Nguyen

**Affiliations:** 1 Department of Behavioral and Community Health School of Public Health, University of Maryland, College Park, 4200 Valley Dr, College Park, MD 20742 USA; 2 Department of Epidemiology and Biostatistics School of Public Health, University of Maryland, College Park, 4200 Valley Dr, College Park, MD 20742 USA; 3 College of Information, University of Maryland, College Park, 4130 Campus Dr, College Park, MD 20742 USA

**Keywords:** Maternal and child health, AI, Artificial intelligence, Community-engaged research, Health disparities

## Abstract

Maternal and child health is widely recognized as a marker for a healthy society. According to the Centers for Disease Control and Prevention, the US maternal mortality rates have remained high, with over 1200 maternal deaths occurring in 2021. Recognizing that systemic racism is embedded at all levels of the public health and medical fields, intervention is needed at all levels of the socio-ecological model. Developing health communication tools for pregnant women and new parents is one leverage point in the numerous changes that must occur across public health and medical fields to achieve maternal and child equity. The current study employed focus groups to inform development of an interactive question-and-answer chatbot called *Rosie.* Participants (*n* = 30) were all pregnant and new mothers of color residing in the United States. Data were collected in virtual focus groups (*N* = 6) and transcribed verbatim. Template analysis of focus group transcripts produced three themes in women’s health information needs and preferences: (1) Pregnancy and New Parenthood Challenges, (2) Sources of Information and Support, and (3) Chatbot Design. Chatbots as a purveyor of health education information were perceived as a promising approach among our pregnant and new mothers of color participants, who had an array of needs that could be addressed by an intervention such as a chatbot. This technology has broad applicability in the health sphere and may serve as an important supplement to both clinical care and existing early childhood intervention services.

## Introduction

Maternal and child health is widely recognized as a marker for a healthy society. According to the Centers for Disease Control and Prevention, the US maternal mortality rates remain high, with over 1200 maternal deaths occurring in 2021 (Hoyert, 2023). The impact of systemic racism on the health of American women of color and their children has been well-documented and has yielded both historical and contemporary inequities in maternal and infant mortality and morbidity (Abuelezam et al., 2022; Crear-Perry et al., 2021; Dagher & Linares, 2022). Recognizing that systemic racism is embedded at all levels of the public health and medical fields, intervention is needed at all levels of the socio-ecological model (Chinn et al., 2021; Kramer et al., 2019; Sutton et al., 2021). New parenthood can also bring forth new health concerns and a need for tailored health information, which often means more frequent interactions with healthcare providers (Henshaw et al., 2018; Verbiest et al., 2018). For patients of color, this need can be complicated by embedded racism within health systems, and create a barrier to care or require higher levels of self-advocacy as a patient to find a provider who is accessible and is actively addressing explicit and implicit bias in their delivery of medical care and patient communications (Gillispie-Bell, 2021; McLemore et al., 2018; Sutton et al., 2021). Prior experiences of racism in patient-provider relationships can result in patients losing trust in the medical system, increased hesitance or delays in seeking medical care, and can increase stress, anxiety, and exacerbate other health concerns during pregnancy (Adams & Craddock, 2023; Altman et al., 2020; Conteh et al., 2022; McLemore et al., 2018). Developing health communication tools for pregnant women and new parents is one leverage point in the numerous changes that must occur across public health and medical fields to achieve maternal and child equity.

Chatbots have been identified as a promising way to address health information needs in a tailored, user-specific way by leveraging recent advances in natural language processing and artificial intelligence. Chatbots have advantages over traditional health communication materials like pamphlets or books which cannot be updated once they are distributed. Chatbots also can be used to address a common issue with web searches for maternal and child health where conflicting information or too high of a volume of information when an answer is needed quickly causes more parental stress and anxiety by providing tailored, concise answers to health-related questions (Kraschnewski et al., 2014; Montenegro et al., 2022; Rathbone & Prescott, 2019). Pregnant and parenting women of color have also expressed a need for culturally tailored advice and communication, something that is often missing from patient-provider interactions (Alexander & Clary‐Muronda, 2022; Chua et al., 2023; Nicoloro-SantaBarbara et al., 2017). Chatbots’ knowledge bases can be developed to inclusive cultural practices and norms about pregnancy and parenting and be responsive to questions about specific practices when developed in partnership with the intended audience (Chua et al., 2023; Perski & Short, 2021). Prior evaluations of the acceptability and feasibility of chatbots for pregnant women and new parents have also indicated that engaging the intended audience for the chatbot throughout all stages of development yields a final product that is rated as more engaging and acceptable (Bickmore et al., 2020; Chua et al., 2023; Guendelman et al., 2017; Perski & Short, 2021; Suharwardy et al., 2023; Virani et al., 2021).

### Current Study

Given this context, our team developed an artificial intelligence-powered chatbot called *Rosie* for pregnant and new mothers of color (Mane et al., 2023; Nguyen et al., 2024). Initial development of the content and features of the chatbot were derived from a series of focus groups conducted with women of color who were currently pregnant or parenting an infant to assess what features and content are most needed and would facilitate chatbot use. The objective of the current study is to qualitatively explore these experiences and needs of women of color during the perinatal period through focus group discussions. This study helps to illuminate women’s informational needs and desires for addressing their concerns, with particular attention to the potential of digital health interventions.

## Methods

### Setting and Sample

The focus groups were conducted and recorded using Zoom with a semi-structured interview guide. The six focus groups (*N* = 6) were led by the Research Coordinator and another member of the research team. Five of the focus groups were conducted in English and one was completed in Spanish. Thirty-two (*n* = 30) currently pregnant women or mothers who had infants under the age of six months participated in the focus groups. Participants were aged 17 to 39 (*M* = 29.1) years. The sample consisted of 43.3% African American and Black women, 23.3% Asian and Pacific Islander women, 13.3% multi-racial women, and 20% white Latina women. Approximately one in three participants identified their ethnicity as Latina. The vast majority of participants had earned a bachelor’s degree or higher (86.7%) and had health insurance (90%). All participants reside in the United States, including New York, Washington D.C., Maryland, California, Texas, Georgia, Ohio, Illinois, and Minnesota. Sample characteristics are summarized in Table 1.

**Table 1:** Participant Demographics

Variable	*n*	% of sample	M *(SD)*	Range
Age			29.1 (5.3)	17–39 years
Race				
Asian and Pacific Islander	7	23.33%		
Black/African American	13	43.33%		
Multi-Racial	4	13.33%		
White Latina	6	20%		
Ethnicity				
Latina	10	33.33%		
Non-Latina	20	66.66%		
Education Level				
High School Diploma/GED	3	10%		
Associate’s Degree	1	3.33%		
Bachelor’s Degree	16	53.33%		
Master’s Degree	5	16.66%		
Doctoral or Professional Degree	5	16.66%		
Household Income Before Taxes				
$0 - $30,000	1	3.33%		
$30,000 - $50,000	1	3.33%		
$50,001 - $75,000	5	16.66%		
$75,001 - $100,000	14	46.66%		
$100,001-$200,000	7	23.33%		
$201,000 or more	2	6.66%		
Number of People Living in Household				
1	0	0%		
2	12	40%		
3	12	40%		
4 or more	6	20%		
Health Insurance				
Insured	27	90%		
Not Insured	3	10%		

### Data Collection

Focus group participants were recruited through convenience sampling by sharing flyers with information about the focus groups and a link to an interest form on Facebook and Instagram. Potential participants completed an interest form in Qualtrics indicating personal information and preferred communication method. Following this, the research coordinator contacted each potential participant and planned a Zoom meeting for each group based on participant availability. During the Zoom meeting, the Research Coordinator explained the purpose of the focus groups and study, how the focus groups would be conducted, expectations when participating, and verified if participants met enrollment criteria. Potential participants were informed within two days if they were eligible to participate and provided a Zoom link to their focus group. Two days before each scheduled focus group, the Research Coordinator emailed each participant a reminder and a copy of the consent form for them to review.

Focus groups were conducted by one facilitator and one co-facilitator from the University of Maryland. At the beginning of each focus group, the facilitator welcomed participants, explained how the focus group was going to be conducted, reminded expectations when participating, and went over the consent form giving participants the chance to ask questions, make comments, and verbally consent. Once everyone consented, the facilitator reviewed a script previously written by principal investigators and approved by the IRB, which included questions about challenges as a new parent, ways to cope, parents’ needs, sources of information they used, and feedback on Rosie the chatbot.

There were a total of six focus groups (*N* = 6), two focus groups for Asian American and Pacific Islander mothers (*n* = 7), two focus groups for Latina mothers (*n* = 10), and two focus groups for Black/African American moms (*n* = 13), for a total of 30 focus group participants. The number of participants in each focus group ranged from three to eight participants, and each focus group lasted approximately 90 min (range one to two hours). The duration of each session varied based on the number of participants per group, as smaller groups progressed through the questions more quickly. Nonetheless, all questions and topics were addressed across all groups. One of the focus groups for Latina moms was conducted in Spanish. Each focus group was audio–video recorded and transcribed verbatim, using participant-selected pseudonyms. Participants were provided a $30 gift card as compensation for time spent during the focus group.

### Data Analysis

Our team analyzed the transcripts using a template analysis approach (Brooks et al., 2015). We started by familiarizing ourselves with the data and created a preliminary codebook using anticipated themes based on the qualitative interview guide. We then used the anticipated codebook to do a line-by-line coding of each transcript (Brooks et al., 2015). Next, we organized the emerging themes into a thematic structure and developed a coding template (Brooks et al., 2015). Finally, we applied the template and finalized it (Brooks et al., 2015). Throughout the analysis process, our team met to discuss the analysis process and adapt the codes and template to ensure that it reflected our interpretation of the data (Brooks et al., 2015). Each transcript was coded by two members of the analysis team.

### Reflexive Statement

Our team is a diverse group of public health, social work, and computer science researchers. We have a wide range of races and ethnicities represented on our team, including African American, Asian, Latinx, and white. Our team is composed of people who have diverse sexual orientations and gender identities. Some of us are parents and some of us are not, and all of us have prior experience working with pregnant and parenting people through direct practice and/or research. As part of our commitment to reflexive practice, we continually reflected on ourselves in relation to the research through both peer debriefing and individual reflexive journaling.

## Results

### Theme One: Pregnancy and New Parenthood Challenges

Participants described a range of challenges and ways to cope with these challenges during pregnancy and their children’s infancy. They described often having questions they were uncertain how to address.

#### Subtheme 1a: Challenges During Pregnancy and New Parenthood

Mothers’ primary challenges included the impacts of pregnancy on their bodies, mental health challenges, identity changes, infant feeding, and decisions about when to handle issues at home and when to seek clinical care. Mothers shared experiences of nausea, vomiting, and back pain as well as significant anxiety and a sense that they were becoming a different person and their body was no longer theirs. Elite described feeling like “I don’t know who I am,” after which Norsly shared similar experiences and commented, “I get sad for no reason sometimes…I just start to cry. I’m like, ‘what is the reason I’m crying?’” Some of the mothers explained that they understood these shifts were due to hormonal changes; however, actually coping with the changes in their bodies and emotions was challenging. As M explained, her biggest challenge of “always being concerned that something was going to go wrong” with the pregnancy led her to withhold information from her social network. The women simultaneously longed for connection and knowing they were not alone while fearing judgment from others, particularly mothers with more experience. The women discussed the impact of miscarriage on their mental health and the importance of normalizing how common miscarriage is. As Sacha explained, “it’s literally your body trying to get ready [for pregnancy]. It’s very common.” Mothers described challenges with breastfeeding, particularly with first babies, and the patience moms need to give themselves and their babies. Lily shared:

Another thing that I struggled with was…breastfeeding, but with my first one, it was the hardest…after we came home. [At the hospital] I showed, like, you know, ‘Oh, I got this.’ And [the nurses] were so proud of me [laughter]. You know, like, [you’re a] first-time mom, and you got this. We came home and I just [couldn’t] get him to latch. We [went] to, you know, the late Super Walmart that's open 24 hours and whatnot at like 3:00 a.m. I'm like, um, ‘It's been so long that the baby hasn’t eaten.’ I just told my husband, just go get formula, you know, he’s gonna dehydrate and everything. So, an important thing is that the baby is fed. … It could be that [my baby] picked up from my tiredness and frustration and that’s why, you know, it didn’t work out, because he did eventually pick up on breastfeeding.

Mothers, particularly first-time mothers, struggled with knowing what to manage at home and what to make an appointment with their medical provider to address. As Niki explained, “my biggest challenge… is to figure out when it is that I need to see the doctor, or when it is not something to worry about and I can just deal with it at home.” Mothers found that searching online often made them more anxious and didn’t address the issue. Samantha shared, “I think when you look on Google you can stress yourself out more. You’d look at the rarest of rarest thing and say, ‘What if this could happen, or what if that can happen.’”.

#### Subtheme 1b: Coping with Challenges During Pregnancy and New Parenthood

To address the challenges experienced, mothers utilized different strategies to cope and feel better. For example, mothers mentioned researching information online, going to therapy, engaging in activities, exercising, doing yoga, and getting support from others. Therapy was a particularly important way to “ease my mind or cool myself down.” Mothers expressed that being first time mothers was difficult because they didn’t know what to expect and how to make sure their babies were safe. Thus, one way to cope with this feeling of uncertainty was by searching online for information about their pregnancy. Pinky said:

Okay. So first of all, when I got pregnant, I mean, my three-year-old child, I was very worried at first because I’ve heard a lot of stories about, like, miscarriages, people losing their baby, people […] giving birth earlier than they are supposed to do, so I was very worried. I was feeling tense. I was very anxious. So I decided that I should just go online, read more, get more information from Google.

Some mothers shared that they struggled with changes in their routine and tiredness. Bee shared drastic changes in her appetite, sharing “I kinda eat a lot now, so I get—I-I’m able to, um, adapt to it by surrounding myself with snacks,” and sleep patterns, “I get sleepy, you know, so, um, I’m trying to get used to it, and I’m trying to, you know, keep myself busy with some activities.” Em commented that in her case “the one challenge that I do have almost all the time is, uh, mood swings” and how exercising and yoga helped her cope: “exercise helps to, like, release certain endorphins that actually helps to elevates the mood”. She also said “Yoga has really helped me a lot in taking control of my moods. Whenever I feel down, I just try and breathe deeply and meditate, and it works.”

Feeling supported throughout their pregnancy was also something that mothers expressed was very important for them. They described how counting on someone to either help them, listen to them, or advise them was something needed to cope with their feelings and challenges. Norsly shared about her husband, “He’s always been there for me, you know, first pregnancy requires a lot of attention because you’re gonna face a lot of stuffs, so he was there for me.” Samantha shared the same experience with Norsly and mentioned how important family was in her coping process. Samantha said, “Coping with it I think having a lot of family help close by was helpful. And then realizing that, uh, I guess I can't do everything.” In Em’s case, talking to her midwife was very helpful: “I just, like, try to talk to my midwife’cause I believe that she knows best about these kind of things. So she gives me tips sometimes” and how her support helped her deal with her pregnancy: “so many other tips she gives that actually works. It really helps to talk with her.” Millie agreed with the feeling of support and mentioned one of her resources: “I join online moms group, that has helped me a lot.”

#### Subtheme 1c: General Questions About Pregnancy and Parenting

Mothers’ main questions about pregnancy and maternity were related to parenting, medication, health, and relationship with their partner. Mothers shared that being a new mother caused them to feel insecure and even depressed at times. Therefore, many of their initial questions were guided toward themselves. Em said, “I was wondering what kind of mother I was going to be while my baby was inside me” she continued. “I was really not ready when I got pregnant.” Elite shared the same feeling as Em and added, “I consulted myself first. Like, I told myself, girl, are you really ready for this?” Millie mentioned that, in her case as a new mother, she constantly questioned if she knew what was best for her baby: “do I really need the best thing—is it safe for my baby,’cause, uh, it’s my first child. I got it, anxiety, this fear in me. I don’t wanna lose my child.”

Questions related to medication were also prevalent, Belen said, “I was getting congested. I thought it was a cold. I thought it was allergies. Um, didn’t really know what medications I should be taking, especially the night ones”. Belen also mentioned, “I was taking prenatal, the daily one, but then, on top of that, I was taking calcium and iron and vitamin C on the side like with it, and I started getting migraines’cause I was probably taking too much of it.” Sarah agreed with Belen and stated that medication was an unclear topic for her. Sarah shared, “I’ve heard you avoid ibuprofen, but acetaminophen is okay, and how much. Um, so that’s been tricky to balance.”

Besides not knowing what medication was ok to take or how much of it, mothers also stated that they struggled with their and their babies’ diet. Em commented that “the issue of what to eat, what to do, and what not to do” was common for her and that she usually had to ask her midwife since she did not know. Sarah shared that she also had many questions about her diet because feeding rules continue to change. Sarah mentioned “sometimes like seafood is okay, obviously, not raw, and stuff like that. Um, and I know a lot of those rules were very strict before with pregnant women, but now you hear that, “Oh, a little bit is okay.”

Health questions were also predominant. Mothers spoke about questions related to unexpected changes in their bodies. Julia shared, “On the third trimester, mostly, you’ll get skin tags, so I was getting a lot of skin tags around my body, and I didn’t know: Where is this coming from? Is it pregnancy-related or not.” Niki shared a similar experience as Julia; Niki said, “ in my second trimester I started getting spotting. I just wanted to know if other people had similar experiences and how that ended for them and stuff.” In J’s case, she had specific questions about her baby’s health. J stated, “In my family, we have, um, a history of diabetes. So, I was really curious about if it was, um, something that would apply to my child”.

Lastly, mothers described having trouble connecting with their partners and wondered if it was normal. Julia explained “a challenge for us has been, um, connecting more like with our partners now that the baby’s here.” Belen agreed with Julia and stated “For a few months, uh, I didn’t want my boyfriend to like touch me. I didn’t want him near me. I was just like, uh—just like, kinda, “Stay away from me.”

### Theme Two: Sources of Information and Support

Participants described where they get information and support about maternal and infant health, as well as what they thought was missing from their current sources of information.

#### Subtheme 2a: Sources of Information

Participants expressed using a number of sources of information regarding parenting, from physicians to family and friends to the internet. They discussed consulting different sources for different types of information and tended to use multiple sources. For medication or medical questions they tended to ask healthcare professionals but relied on family or the internet for other kinds of information. For example, J discussed how the source depends on the questions:

Many questions that I think I can find online, I quickly google it, but on more intricate questions that I don't think would have been explained properly, I make sure to ask my GYN-y. She—I remember the question about diabetes. I had questions about sex.

Alexa described asking the doctor for more serious questions but “secondly, I-I'll move to my mom, if—you know, if it's a minor problem, I usually ask my mom. And if it's something I can search on Google, then I use Google.”

Some of the participants did limit how much they asked their providers out of fear that they would annoy them or due to lack of time. Niki asked anyway, but was concerned about annoying hers: “I would directly ask, for example, if I had any issues with OBGYN/pediatrician just to—just—even if it annoys them and I have to ask them a multiple times it is—I’d rather be safe than sorry.”

Unlike some of the other participants, Nita was able to rely on her provider for most of the information that she needed:

Even when I was actually experiencing some kind of emotions, my feelings, changes, and all the hormonal stuffs. She was actually there for me. Like, I could go to her. If I called, sometimes we'll just set up some kind of Zoom meetings where, like, we just do some kind of video calls and all that. So, she was basically there for me. And whatever I was kind of experiencing, she was just there to givin' the right answer at any time.

Many of the participants relied on their family or friends with experience for advice about pregnancy and postpartum care. Julia used her family’s practices to guide her intuition:

For me, aside from speaking to a doctor or to a family member, I will—I’ll talk to a friend or recall how—something that my mom used to do when I felt a certain way. Like when I was sick, what did my mom used to do? Um, and so I tend to go to that or a friend, or sometimes it’s, I guess, the motherly instinct, something that you just know that feels right or doesn’t feel right. Sometimes we just simply have to trust our bodies and how we feel.

Most of the participants used online sources for reassurance and information. Instagram, Facebook, BabyCenter, Ovia, medical websites like Mayo Clinic and WebMD, and Google searches were all frequently used. Online forums or groups where mothers could share information or get help were valued as “tools to deal with all of it” (Emily). Camila used Instagram and followed “many pediatricians, followed many obstetricians and gynecologists, and there I could resolve my doubts.” M used the Ovia app “for what foods to eat, what medicines to take, and then what is happening inside my body at this point.”

Getting in-depth and complete information was reassuring and helped participants feel like they could parent better. Alexa used websites for very detailed information:

Well, I look for more detailed information. … I don’t just go to…some websites [that] just, you know, give you the easy answers, but I look for the ones that, you know, give me details about what I’m lookin’ for.

J wanted to get things right as a parent:

I engage reading a lot of books about, um, being a parent because I really didn’t wanna do anything wrong. And then I also use Google. I am a big fan of that. Anything I don’t know, I’m quickly googling it. And then sometimes I use my Siri to search. It’s much easier and saves a lot of time, so I guess I search things I do not know or understand.

#### Subtheme 2b: Missing from Sources of Information

Although participants had many different sources of information, there was variability in how helpful they were. For some of the participants, the amount of information and feeling like they had to consult multiple sources led to information overload. Niki was unsatisfied with the lack of complete sources and needing to check multiple places:

There are different articles on different topics, but there’s no place that cohesively has everything that I need. So like, search five different websites to get the answer. Like, if everything was in one place, that would be really helpful.

M described: “I find a lot of answers, but they're not satisfying answers. They’re not—or not useful or, like, what do you do about this?” Pinky agreed: “even though Google is widely-known and it provides a lot of information, um, sometimes you don’t really get the answers to what you want.”

Participants worried about the reliability of certain sources, particularly what they found online. Sarah shared her concerns: “so much you have to filter through when you’re on Google. Um, it’s not—it can’t always be super reliable.” The participants felt that finding reliable sources created extra work for them. Julia described:

You need to do some research to be able to find the answer that you need, but not only—sometimes even if that is the final answer, but can you really trust it? Is it really what it is? Because it’s not necessarily coming from a doctor, from, like, a scientific research, and that’s why apps and stuff like that make our life easier ’cause then you don’t have to read a whole scientific article about it… But - but, yeah, oftentimes, we - we do have to make some extra research and ask somebody just to confirm about it.

The participants also mentioned that the information they found online sometimes caused more stress. Samantha shared: “I wish there was something putting your mind at ease. Which I think when you look on Google you can stress yourself out more.”

Another frustration with current sources that was voiced was a lack of practical information. Participants wanted to be able to find out exactly what infants need, where they could buy maternity clothes, what size diapers and clothing to stock up on and how to deal with things like postpartum hair loss. Em wanted information on how to get her baby health insurance and Nobu Powers felt that information about budgeting would be helpful. The participants also expressed a need for information about birth control options and sensitive topics like sex.

One of the biggest concerns that participants had about current sources was the lack of formal or professional support. Many of the participants wanted to have more time with their doctors or better access to information from a medical provider. Some of the participants paid for services that gave them access to a doctor through a mobile app but still expressed dissatisfaction with the availability of the providers. NR wanted more time with her provider and to have more personal care:

The OB visits are so short. It was crazy ‘cause I went a lot like a couple of weeks ago, and I talked to the doctor for literally eight minutes. And for me, that just felt so strange that I waited for you for longer. But, you know, take—doing the urine thing was longer. But talking to you—it just felt very disconnected to me that, um, I felt I wanted more reassurance. I mean, as much as it sounds crazy, I just want him to be like, ‘You’re okay. You’re doing okay,’ rather than it being so mechanical. And, ‘Oh, let’s check your heartbeat. Okay, you’re good to go. You’re not swelling. Bye-bye. You know, see you in-in four weeks [or whatever] two weeks.’ And I think I would love to have—and that’s when my mind kinda started going, ‘Should I look into a doula? Is that what I’m looking for? Do I need a midwife?’ It’s just no one really talks about it, so I have no idea which direction I should go. I can Google things, but who-who can answer these questions, you know. And is the OB not right for me? He seems fine.

Finally, the participants wanted support and reassurance from the sources of information. Julia said: “I think the most important thing you need is support, someone that will always be right next to you saying, ‘You’re doing fine. You’re a great mama, and you’re doing wonderful.”

### Theme Three: Chatbot Design

Participants were excited about using a chatbot to address their pregnancy and parenting questions. We solicited information both about the apps they already use and what kind of features would be important to include specifically for a chatbot.

Participants were asked about their current phone usage and their preferred apps for both general use and information specific to pregnancy and parenting. Most participants reported frequent use of WhatsApp, a chat app, for both personal and professional reasons. Facebook was more often used for keeping in touch with friends and families and engaging with groups, including parenting groups. TikTok and Instagram were frequently mentioned as sources of entertainment. Participants also discussed health apps they were currently using, with menstrual period and fertility tracking apps like Clue or My Days being the most commonly used. The most commonly mentioned app for pregnancy and parenting was What to Expect, which users used in both English and Spanish. One participant who was pregnant at the time of the focus group, Nobu Powers, described her usage of What to Expect as “at this point all I really use it for is to see what size my baby is. Like today it's the size of a prune or olive. So that's the extent that I'm using it right now. And then maybe some one-off articles about, like, nausea at this stage or, you know, what not to eat.”

Participants often spent a fair amount of the discussion groups looking at and making recommendations for the chatbot’s design. Both ease of use and overall aesthetic appeal were emphasized as being important and motivating usage. Data security and privacy were also very important to participants.

#### Subtheme 3a: Information Quality

Participants also emphasized the importance of having sources for the information provided to establish credibility and accuracy. One participant, Samantha, expressed, “does it say who's giving the advice? Is it by, like, a-a health professional, or like moms or somebody with more experience like a midwife? Like, it doesn’t specify where the knowledge is coming from.” Samantha also noted that having links to source materials would also help with getting a deeper understanding of the topic. “I think if you had, um, like a link [from *Rosie’s* response] to [the source documents] if you wanna read further about this topic.”

Julia noted that having video examples of certain tasks, especially if they involve many steps, would make using the app easier by getting directly to the solutions instead of having to read a large block of text first. For her, having brief video examples was “more dynamic” and “would be better than reading a long, long answer—because I don’t know—depending on how you typed your questions, how specific *Rosie* is going to be”.

Sasha also noted that having questions built in to rate the quality of the responses was important to her based on prior experiences with chatbots: “If I’m chatting, then I would like the prompts because I’m used to it in different avenues. That’s usually how the AI is being built. Um, like, even if you go to, like-like, you know, a-any site, like even in your workplace, like, did I answer your question? Like, would you like to know more? Like, do you want more resources? And they’re like—they’ll, like, offer you those choices. And then at the end, it would be like if I’ve answered all your questions, like, you know, do you wanna end this conversation?”.

#### Subtheme 3b: App Functions and Preferences for Customization

**Fig. 1:**
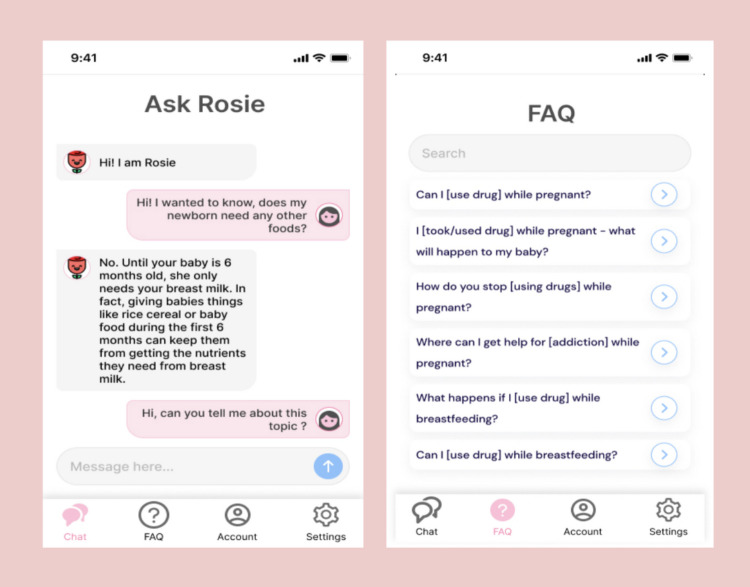
Rosie prototype screen displayed to focus group participants

Many participants valued simplicity in design as it helped with the ease of navigating the application and indicated simpler designs were more aesthetically pleasing. Nobu Powers described the app prototype (see Fig. 1) as “cute and simple.” J also mentioned, “I really have issues where apps tend to be designed too complicated. I like things simple where I can ask the questions, not too many controls, and, if possible, it should be divided into as little subsections as possible.”

Participants had varying ideas of how the app could operate to be most useful and preferences varied. Em requested that having built-in reminders to the app would be helpful: “ I think it would be really cool if an app was, like, assigned to, um, remind expectant mothers to take their supplements, to exercise, to not forget their appointment, and—because I—personally, I forget things a lot at-at this stage. I forget things a lot. That is why I have to, like, set reminders across my calendar,” while Sarah reported finding too many notifications made her stop using another pregnancy app: “I had found this one app, uh, that kinda went step by step, like every week, what’s new, what’s happening to your baby inside. Um, but then it got kind of annoying. Like it would send me lots of notifications and stuff, to the point that I didn’t end up using it, and I was trying to find something new that would give me the same information”.

As many products for soon-to-be parents are often marketed using gendered colors, there was a similar preference expressed by participants. Participants expecting boys wanted to change *Rosie’s* display colors from pink to blue. Belen said, “Uh, why’s it all pink? I mean I get that the name is *Rosie*, but I’m expecting a boy, so why [laughter]—why is it all pink?” when looking at the app prototype. Pink noted that while she has a preference for the color pink, the ability to customize the look of the app was important: “Okay. So, um, I think this color is great, as I said earlier on, but there should be a feature where, um, I should be able to change the colors. You know some people prefer, like, blue to pink, so there should be something which, like, maybe a feature that maybe changing color, change background, something like that. So for instance, if I give birth to a girl, I can use this. If I want to track the dates of a boy, I will use the blue, and that makes it more attractive.”

Participants also noted they had preferences for designs that integrated more representations of people rather than simple static icons, with participant M stating “It might be nice to have a face that looks more human just ‘cause you’re potentially sharing personal information with this chatbot” and Sasha expanded this by explaining that customizability with her own photos made using *Rosie* feel more personalized, “I would like to expand on what participant M said about the make it more personable on the, um, on the face, um, the picture of *Rosie*. So, I’m-I’m going back to, like, you know, when I-when I open Instagram, like, it’s my page. Like, it's me, and it's my photo, so it's then, like, I know, like, this is—so maybe something to do with *Rosie* as an icon and my photo that you upload like your own profile or, like-like, you want the user to come back and click on the app and be like, “Oh, it’s-it’s, like, my thing.” Like, your medical record is there, so it’s like you feel that it’s your page, um, your app.”

#### Subtheme 3c: Specific Questions for Rosie

Participants’ questions for *Rosie* focused on both their health and the health of their fetus or infant. Participants were concerned about their own mental health, diet and exercise, and understanding medication and supplement safety during pregnancy. The focus on these topics specifically may also reflect the fact most of our participants were pregnant at the time of participation.

Nita talked about her experiences with sudden bouts of sadness and crying and wanted to ask *Rosie* if these experiences were normal and how they could be managed. “ I just wanna say I would love to ask *Rosie* on a basic way on how to deal with my emotions because I could remember when I was pregnant from my first daughter, like sometimes, I get been—like I get emotional. Sometimes I just get sad without no reason. And I start to cry. I was like, why am I crying? And [laughter] ther-there’s no reason why am I crying. So I-I would tend to ask, um, *Rosie* on how to deal with those emotions when it comes around, so maybe how to get happy when I’m said, stuffs like that.” Samantha had a similar question to understand the differences between expected changes in mood and what could be more serious, wanting to know “what are ways to know, like, this crying is normal versus there’s something you need to get checked out?”.

Mothers sought tailored advice on appropriate weight gain and weight management along with a focus on the nutritional quality of their diets. Belen expressed “But then, all right, so I’ve gained my, what is supposed to be the norm, 25 to 30 pounds?. Do I change my diet? Uh, what am I to do now? What do I cut down? Do I cut down? Like I’m not sure where I am nutrition, uh—nutrition-wise—nutrition-wise,” and other participants expressed similar concerns about the lack of specific guidance for their diets, with Carla asking “¿Cuál es la mejor nutrición durante el embarazo y de acuerdo a los meses?” (What is the best way to eat [for nutrition] for each month during pregnancy?). Participants were interested in getting more personalized guidance on exercise and weight management during pregnancy. Norsly noted that sometimes recommendations she received during her first pregnancy did not always match her current abilities and getting tailored guidance would be reassuring: “Yeah, it’s very, very important on what exercise you ought to participate in because I could remember when I was pregnant with my first child, and I was asked to run, uh, eight meters, and I got tired when I just ran, like, two meters, and I was, like, wow. This kind of exercise is too much while you’re pregnant. So this situation, like, I will want to know what kind of exercise I should engage on, what kind of sleeping patterns, you know, what should I eat, and all that-that kind of affects my decision.”

### Theme Four: Facilitators and Barriers to Chatbot Use

Participants readily identified common issues they had with other apps they had used, what they felt were the most useful utilities a chatbot could provide, and again emphasized the importance of the chatbot reducing the cognitive burden of sorting through information, making comparisons, and concluding which information is the most-up-to-date and accurate for their question.

#### Subtheme 4a: Speed, Accuracy, and Conciseness

Participants were thorough in articulating what they would dislike in an application and what could dissuade them from using it. Participants prioritized quick and accurate responses that saved time, as Samantha described, “cause, what I don’t want is that whole time that on my phone I’m tryna, like, step back from my everyday life that I don’t want my phone to remind me of that.” Other applications that used videos to provide information or examples were sometimes not concise enough for participants, as Nobu Powers explained “the apps that I’ve been using, a lot of them they—there’s videos. And then sometimes you just—you don’t wanna watch a whole video”. NR noted her preference for video demonstrations but also emphasized the importance of brevity, “I love that and is it too much to ask your app to also maybe include, like, video tutorials on certain things? I mean, like how do you change a diaper? You know, I-I—now I feel like this is high expectations, but, you know, it’ll be nice to see a quick resource and this is how you change a diaper and, um, even image demonstrations or a video link.” NR also explained how a well-designed chatbot would be extremely helpful “honestly, when I was looking at this question and I saw the answer I thought I would love to have this right away just because I don’t have, you know, I would do that on Google, but there’ll be so many different answers, but just someone kinda gave me that—in general, like, the concise, quick, just get to the point, um, kinda answer. This is—this seems helpful, so very likely I would love to use something like this.”

*Rosie’s* ability to understand users’ questions and generate useful responses was also important to focus group participants. M discussed this at length:

“I didn’t think about the language and also the-the jargon that, like [laughter], younger—like different people—you know, or like or even language barrie—like language barriers not knowing exactly how to spell things. Like, I-I wonder if, um—I wonder if—like what does *Rosie* do if she doesn’t understand a question? Like, in that case, can she prompt—like, is this what you mean or something like that? Or do you have a way even to make it available in different languages, or that’s more the language. [Laughter] It doesn’t really capture the for real issue [laughter] but-but is there a way for it to kind of say like, "I’m not sure I understand. Is this what you’re trying to ask me?" And then give prompts, so the person still can express themselves the way they want to, and then they maybe realize, like, I have to be more clear about what I’m saying or-or are given options.”

Sasha noted similar thoughts:

“I was thinking of that when you were talking about, um, like the prompt versus the non-prompt because, um, I-I’ve seen the S—uh, the Facebook groups of, like, different moms asking different types of questions and, like-like, some people just type, like, and I-I don’t know what you call it, but it’s-it’s not like—it’s like truncated English. Then I have to, like, understand what they’re saying. It’s like FR. Oh, for real. Okay. And then I have to, like, think back on my brain of, like, the emoji languages and what they’re asking. So then in those cases I feel like if *Rosie* were to prompt them, then they kind of can get their an—like, question right because some of—like literally some of the questions on the Facebook groups, and they’re just, like-like, it’s hard for me to understand personally. Oh, I’m just probably dating myself now [laughter] but, um, but, you know, I’m not sure *Rosie* can understand that co—like, colloquialisms and all of that thing, but, like, you know, are you expecting everyone to type really nice English? You know, full sentences. You know, question mark. You know, this is what I want to answer today.”

#### Subtheme 4b: Customizability and Congruence with User’s Values, Experiences, and Parenting Decisions

The variability in the experience of pregnancy and parenting and having the chatbot recognize and respond to specific needs and values was emphasized repeatedly by participants. In thinking of ways to design the chatbot as effectively as possible, Nobu Power stated that “if there were specific categories that catered to, I guess, their specific situation. That would definitely—like, making the chatbot not as generic. Like you can choose—like I think somebody said before. Like if you have a preemie baby, then you can, you know, categorize that yes, I have a preemie baby, and this is the issue. Or, you know, I’ve had, you know, this many miscarriages and, like, this is my rainbow baby. Things like that that would, you know, kind of make you feel like yeah, I’m visible. The app knows that there’s people like me in the situation and, um, you know, it has answers for me.” Samantha also noted that parents of children with specific health needs may not view their needs as being met by a chatbot unless the app could demonstrate its ability to customize responses to a variety of health circumstances: “I would think that people who wouldn’t be comfortable with it would be, um, people who, like, have kids who are a little bit different because of medical reasons. They’re preemies and all kinds of other things that I feel like unless it’s that special section, um, for that group of people. You know, kids with, uh, congenital, uh, like, heart problems and stuff like that. It’s kind of hard to—like, I know it’s kind of a general audience. Um, so I feel like when kids—when parents think that their kids are a little bit different for whatever reason it may be. That might prevent them thinking “Oh,” you know, “but my kid is not like this. My kid’s different.”

Variability in the willingness to trust and view certain medical sources as credible and safe was also mentioned. Samantha discussed how her experiences may contrast those of people of other backgrounds: “cause I feel like depending on what community you come from— how much they trust the healthcare is different. So, I feel like—and maybe I’m generalizing here when I’m saying this. But South Asians tend to trust, um, the physicians or health professionals more than some of other communities. I think that’s just how we were raised, um, and the experiences we had as a community. We tend to trust our doctors and our nurses more than other groups who have had more traumatic experiences historically.” Sources or answers that contradicted cultural norms were also potential challenges, as Sarah noted: “whether you’d use home remedies or all of these old wives’ tales that are like part of your culture, um, versus my mother-in-law is like totally fine with lots and lots of medications and this and that. Um, so there’s this kind of—I know that they are on different sides of the spectrum, and so I feel like, culturally, I would want to look at something that seems a little more objective and research-based and stuff, which would be this app”.

Endorsement or recommendation of using the app from other parents or a healthcare provider was frequently mentioned as being important to giving the app credibility. Niki mentioned “if a healthcare professional kinda mentions that this is a good resource to use. I think that will push moms to use the chatbox more,” and Nita described, “I would actually use the app when it is recommended from my family health care provider or maybe, uh, there are, kind of, reviews because I’m a person that goes for reviews. If there are….and also, uh, like testimonies of others, like if they’ve used it, they’ve tried it, and it actually works for them, I think it’s kind of—it’ll be like a motivation”.

## Discussion

Study results indicate that there is a significant opportunity for apps like *Rosie* to serve as a trusted source of highly tailored health education information that can be a complement to clinical care and to efforts to improve culturally competent medical practice with women of color in the peripartum period. Focus group participants were able to provide precise and detailed responses indicating their specific information needs and the deficits they have found in using traditional online searches or parenting apps. The participants found the perinatal period challenging, and how the uncertainty they felt around pregnancy symptoms and learning to care for their infant made it more difficult. Participants’ coping methods included searching for information online, relying on their social support networks, and exercising, which aligns with findings from prior research (Alio et al., 2022). Additional health information and being informed can help mothers deal with uncertainty and better communicate with their doctors (Alio et al., 2022).

The participants used various sources and channels to learn about pregnancy and infant care, including their medical providers, family and friends, and searching for information online. Participants discussed being both overwhelmed with information and having an information gap in some areas. They were not satisfied with the access to healthcare providers which aligns with previous research on the needs of women of color in medical practices (Altman et al., 2020). Women sought additional time with providers, relationship-building, and support in medical decision-making (Altman et al., 2020; Henshaw et al., 2018; Verbiest et al., 2018). In addition, participants noted that trust in medical systems would vary by experience/historical issues (specifically mentioned South Asian women vs. other groups), which is commonly mentioned in the literature about medical mistrust (Gillipsie-Bell, 2021; Sutton et al., 2021; McLemore et al., 2018). More frequent interactions with providers and providers sharing health information can help increase trust and reduce anxiety (Alhalel et al., 2022; Nicoloro-SantaBarbara et al., 2017).

Across all focus groups, participants had concerns over information accuracy and quality and explained the burdens of searching for information and evaluating conflicting information for a definitive answer. Concerns over information quality and stress and anxiety due to contradictory information online or in parenting apps are common findings from prior qualitative research with new and soon-to-be parents (Barnett et al., 2022; Henshaw et al., 2018; Kraschnewski et al., 2014; Rathbone & Prescott, 2019). Recognizing the mental health impacts of poor quality or conflicting advice, it is critical that the development of chatbots for maternal and child health centers on addressing both the problems with current search results and reducing the burden of searching for information. This is particularly important for women of color who are balancing the increased stress associated with racism across the lifespan; they are contending with ensuring the information online is not only generally accurate but is drawn from research and clinical guidance that includes participation from and consideration of mothers with racial and other backgrounds like theirs.

Participants requested apps that could help focus or remove the stress of determining accurate information between conflicting sources and felt that a chatbot based on verified and evidence-based information would be helpful. They also expressed feeling comfortable and interested in using a phone app to search for health information, which aligns with findings among people with higher incomes, yet there is room to evaluate or think of better ways to engage lower-income families or how to address barriers to using a phone as a primary tool for health information seeking (Acquavita et al., 2019; Lau et al., 2023; Jaynes et al., 2022; Jongsma et al., 2020).

Participants appreciated that *Rosie’s* design was simple and aesthetically pleasing but requested the ability to personalize the graphics and number of notifications and wanted the app to address specific needs, including personalized weight gain and specific medical needs. These requests highlight the lack of mobile apps that are inclusive both in visually representing people of color and including culturally appropriate information (Cheng et al., 2020; Tucker et al., 2021). Many currently available apps also do not have high usability, accurate information, or consider consumers’ health literacy levels (Cheng et al., 2020; Tucker et al., 2021). In addition, there is a need for apps that are appropriate for multi-lingual and low-income women as current apps may be contributing to the digital divide (Hughson et al., 2018).

### From Insight to Implementation

**Fig. 2:**
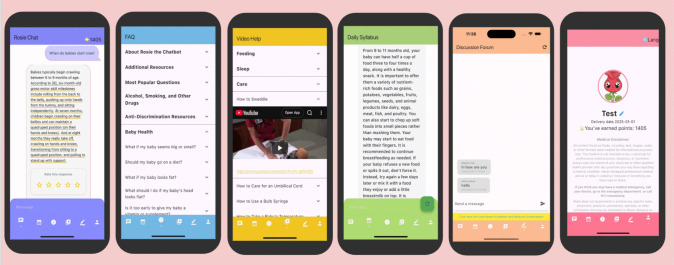
Key interactive features of Rosie. This figure displays the core modules designed to support user learning and engagement, including the Rosie question and answer page, FAQ, Video page, Daily Syllabus, User Discourse Forum, and Profile page

The development and implementation of *Rosie* was informed by the insights we collected from the focus group participants. In addition to the question–answer chatbot, Rosie includes a structured help system with a categorized FAQ page and a video help page to create a more comprehensive user support platform. Based on the baby’s birth or due date, Rosie also sends users daily push notifications that include timely health tips related to maternal and infant well-being. Finally, the Rosie application features a discussion forum where users can discuss and share information with one another (see Fig. 2).

### Limitations and Conclusions

This study is limited by several considerations. Chatbot development is an iterative process and participants viewed a singular early-stage prototype. Second, the majority of our participants became parents or were pregnant during the first three years of the COVID-19 pandemic and may be more open to using an app versus traditional in-person intervention, as these types of interventions were likely the only type offered by their provider or insurer. Finally, the sample was recruited online through social media and the vast majority of study participants had completed college. Already using digital sources of information given their use of social media and higher education levels may mean suggestions for app content and needs could be different among other groups such as those who are not already using social media, younger parents, or those who did not attend college.

Chatbots as a purveyor of health education information were perceived as a promising approach among our pregnant and new mothers of color participants, who had an array of needs that could be addressed by an intervention such as a chatbot as a standalone intervention or embedded in an app like *Rosie*. This technology has broad applicability in the health sphere and may serve as an important supplement to both clinical care and existing early childhood intervention services. This is particularly relevant for women of color in our efforts to move towards health equity given their experiences with suboptimal clinical care in increased stress in the peripartum period. With regard to application within the socio-ecological model, chatbots will never be a replacement for clinical care. They will also never remove the continual need for training of clinicians in culturally competent practice or broader policy interventions to address health equity needs. Rather, chatbots can be a useful tool paired with face-to-face health education and clinical intervention as a highly scalable individual level intervention for anyone with a smartphone. Even for those without one, chatbots can be used by anyone with internet access either personally or in their community as they can be embedded within websites.

In our own development of the *Rosie* the chatbot app, we have incorporated a number of recommendations from focus group participants prior to testing the intervention in a randomized controlled pilot and full randomized controlled trial (Mane et al., 2023; Nguyen et al., 2024). We specifically addressed concerns about the accuracy of chatbot-delivered information by using an underlying knowledge corpus limited to vetted sources (rather than the entire internet, as other chatbots use).

The current Rosie model architecture follows a three-step process consisting of a Retriever model, a Reranker model, and a Reader model. The Retriever model (facebook/contriever), a lightweight and efficient encoder, identifies the most relevant passages in the corpus to address user queries. The Reranker model (TART), a more powerful model, then re-evaluates the relevance of these passages and ranks the top ten. Finally, the generative Reader model (LLaMA) reviews the top-ranked passages to extract the most pertinent information and synthesize a response contextualized to the user’s query. Although the underlying language model (LLaMA) was pretrained on external data, its responses are constrained by the Retriever and Reranker models, which ground the chatbot’s outputs in our vetted corpus.

As a safeguard and added measure for reliability, every Rosie response includes links to specific citations and resources. A two-tone highlighting system in each response provides a visual indicator of how closely each cited passage aligns semantically with the content in the source document, serving as a measure of how well Rosie's response is grounded in the cited sources.

We have covered the breadth of topics the mothers requested during focus groups in both the underlying knowledge corpus and a frequently asked questions tab in the app. We have included a video library to address key frequently asked questions. To address users’ requests for a more customized experience, we implemented a daily notification system. Users receive personalized daily notifications containing health tips and messages tailored to their due date or their baby’s birth date. These notifications include reminders and guidance aimed to help promote positive maternal and infant health outcomes and behaviors including reminders for upcoming well-baby visits, key developmental milestones, and other general motivational messages. Furthermore, we chunked large documents in our corpus into concise, focused, and meaningful passages to prevent long-winded and hard-to-understand responses. This enhances model accuracy and promotes comprehension for Rosie users. Finally, we are testing Rosie as a bilingual Spanish/English app which will allow users to easily toggle between English and Spanish versions of Rosie based on their preferred language.

While chatbot technologies hold great promise for narrowing existing gaps in healthcare, they are with limitations. It is important to remind potential users that these tools are meant to supplement medical care, not replace it. Developers should remain cognizant of existing shortcomings related to AI-powered chatbots, including risks of bias, misinformation, and model errors. Empowering users with transparency about these potential risks and informing them about the sources of the chatbot’s information is a good place to start. Additionally, while chatbots can be useful and feasible resources to advance health, they can only reach people who have access to this kind of technology. As a result, they could exclude users that may need the resources the most but might not have access to technologies like Rosie.

In summary, this is an exciting time as the public use of chatbots has grown exponentially, highlighting both opportunities and challenges with leveraging this technology to improve maternal and child health equity through tailored health education as part of a broader application of the socio-ecological model.

## Data Availability

Data are not available outside of the study team.

## Declarations

### Clinical Trial Number

NCT06053515
